# ﻿First report of the Afrotropical genus *Securiops* Jacobus, McCafferty & Gattolliat (Ephemeroptera, Baetidae) from Southeast Asia, with description of a new species

**DOI:** 10.3897/zookeys.1157.99642

**Published:** 2023-04-05

**Authors:** Thomas Kaltenbach, Sirikamon Phlai-ngam, Chanaporn Suttinun, Jean-Luc Gattolliat

**Affiliations:** 1 Muséum Cantonal des Sciences Naturelles, Département de zoologie, Palais de Rumine, Place Riponne 6, CH-1005, Lausanne, Switzerland Muséum Cantonal des Sciences Naturelles Lausanne Switzerland; 2 University of Lausanne (UNIL), Department of Ecology and Evolution, CH-1015, Lausanne, Switzerland University of Lausanne Lausanne Switzerland; 3 Department of Biology, Faculty of Science, Khon Kaen University, Khon Kaen 40002, Thailand Khon Kaen University Khon Kaen Thailand; 4 Department of Veterinary Biosciences and Veterinary Public Health, Faculty of Veterinary Medicine, Chiang Mai University, Chiang Mai 50100, Thailand Chiang Mai University Chiang Mai Thailand

**Keywords:** Biogeography, COI, eggs, mayflies, taxonomy, Thailand

## Abstract

Recent collections in Thailand revealed the occurrence of the genus *Securiops* in Asia, formerly known from the Afrotropical Realm only. A new species of *Securiops* is described and illustrated based on larvae and eggs. Eggs of this genus are described for the first time. Morphological differences between the new species and the species from Africa are discussed. The number of species in the genus *Securiops* is augmented to five.

## ﻿Introduction

Southeast Asia is one of the regions with the highest diversity worldwide in general, also for mayflies. Much effort has been done in the past years to get a better insight into this yet strongly understudied fauna, including studies of the lesser known, but most diverse mayfly family Baetidae. Emphasis was put on the archipelagos of Indonesia and the Philippines, and continental Thailand. As a result, new genera of Baetidae and many new species were discovered in this region (e.g., Gattolliat 2012; Sutthinun et al. 2018; Suttinun et al. 2020, 2021, 2022; Kaltenbach and Gattolliat 2019; Kaltenbach et al. 2020a, b, 2021; Kluge 2020a, b, 2022; Suttinun 2021; Phlai-ngam et al. 2022; Tungpairojwong et al. 2022; Boonsoong 2022), and more collection efforts and studies are ongoing.

Baetidae are the most diverse family of Ephemeroptera in number of genera (> 118) and number of species (> 1160) worldwide, comprising approximately one third of all mayfly species (Sartori and Brittain 2015; Jacobus et al. 2019; updated by authors). Their distribution is cosmopolitan, with the exception of New Zealand and Antarctica. With continued collections in Southeast Asia and other poorly studied regions with high diversity like New Guinea or the Indian subcontinent, we may expect further new genera and a high number of new species from these regions.

The genus *Securiops* was described by Jacobus, McCafferty and Gattolliat (2006). The type species *S.macafertiorum* (Lugo-Ortiz, 1996) from South Africa was formerly described in the genus *Potamocloeon* Gillies, 1990 (Lugo-Ortiz and McCafferty 1996). Further species are *S.mandrare* Jacobus, McCafferty & Gattolliat, 2006 from Madagascar (formerly described by Gattolliat 2003: 7 as *Potamocloeon* sp. A), *S.megapalpus* Jacobus, McCafferty & Gattolliat, 2006 from Ivory Coast, and *S.mutadens* Jacobus, McCafferty & Gattolliat, 2006 from Gambia, Guinea and Ivory Coast (larva misidentified and described as *Potamocloeondentatum* by Gillies 1988: 53) (Jacobus et al. 2006). Kluge (2020b) proposed *Securiops* as a subgenus to *Procloeon* Bengtsson, 1915, together with *Oculogaster* Kluge, 2016, *Pseudocentroptiloides* Jacob, 1987 and *Monilistylus* Kluge, 2020. They are all sharing the following autapomorphy: one large, posteriorly directed spine outside laterally on each cercomere in the distal part of the larval cerci; usually, it is spindle-like and thickened and its length exceeds the length of the cercomere (Kluge 2020b). However, we are treating *Securiops* as a separate genus in this study, based on a unique combination of characters, which distinguish it from all other Baetidae genera: (1) labium with strongly reduced glossae, enlarged paraglossae, and very broad, hatchet-like palps; (2) tergalii I–IV with two lamellae; (3) legs elongate, with relatively few short setae on dorsal and ventral margins; (4) claws very elongate, without denticles; and (5) lateral margins of posterior abdominal segments with sharp spines (Jacobus et al. 2006). The imaginal stage remains unknown (Jacobus et al. 2006; Kluge 2020b).

Based on the above-mentioned species, *Securiops* has a widespread distribution across the Afrotropical region. Here, for the first time, we report the presence of this genus additionally in Southeast Asia, based on the discovery of a new species in Thailand, which is described and illustrated in this study. We also provide the first DNA barcode for *Securiops*.

## ﻿Materials and methods

The larvae were collected in 2017 and 2019, and preserved in 70%-96% ethanol.

The dissection of larvae was done in Cellosolve (2-Ethoxyethanol) with subsequent mounting on slides with Euparal liquid, using an Olympus SZX7 stereomicroscope.

The DNA of some specimens was extracted using non-destructive methods allowing subsequent morphological analysis (see Vuataz et al. 2011 for details). We amplified a 658 bp fragment of the mitochondrial gene cytochrome oxidase subunit 1 (COI) using the primers LCO 1490 and HCO 2198 (Folmer et al. 1994, see Kaltenbach and Gattolliat 2020 for details). Sequencing was done with Sanger’s method (Sanger et al. 1977).

GenBank accession numbers are given in the Material examined section.

Drawings were made using an Olympus BX43 microscope.

Photographs of larvae were taken using a Canon EOS 6D camera and processed with Adobe Photoshop Lightroom v. 5 (http://www.adobe.com) and Helicon Focus v. 5.3 (http://www.heliconsoft.com). Photographs of body parts of the larvae were taken with an Olympus BX43 microscope equipped with an Olympus SC50 camera and processed with Olympus (recently Evident) software Cell Sense v. 1.3. All pictures were subsequently enhanced with Adobe Photoshop Elements 13.

The distribution map was generated with SimpleMappr (https://simplemappr.net, Shorthouse 2010). The terminology follows Hubbard (1995) and Kluge (2004).

### ﻿Abbreviations

**KKU-AIC**Khon Kaen University, Aquatic Insect Collection (Thailand);

**MZL**Muséum Cantonal des Sciences Naturelles, Lausanne (Switzerland);

**VMCMU**Chiang Mai University, Museum of Veterinary Medicine (Thailand).

## ﻿Results

### ﻿ Securiops primasia sp. nov.

Taxon classificationAnimaliaEphemeropteraBaetidae

ECB598D2-A7B1-5B39-9F18-3AB524185B5A

https://zoobank.org/CD408527-25B4-4F63-BD52-32AFEECF3514

Figs 1 , 2 , 3 , 4 , 5 , 6 , 7 , 8

#### Differential diagnosis.

**Larva.** The following combination of characters differentiate larvae of the new species from other species of *Securiops*: (1) maxillary palp segment II ca. 0.7× as long as segment I (Fig. 3a, d); (2) maxilla ventrolaterally with two groups of simple setae (Fig. 3e); (3) femur approx. twice as long as tibia; tarsus approx. 1.4× as long as tibia; claw approx. 0.7× as long as tarsus (Fig. 5a); (4) hind protoptera absent; (5) tergalii on abdominal segments I–VII, each with two lamellae (Fig. 7b); (6) abdominal segments VIII and IX with large lateral spines, segment VII with minute lateral spines (Fig. 6a); and (7) paraproct with four large, pointed spines (Fig. 7a).

#### Description.

**Figure 1.**
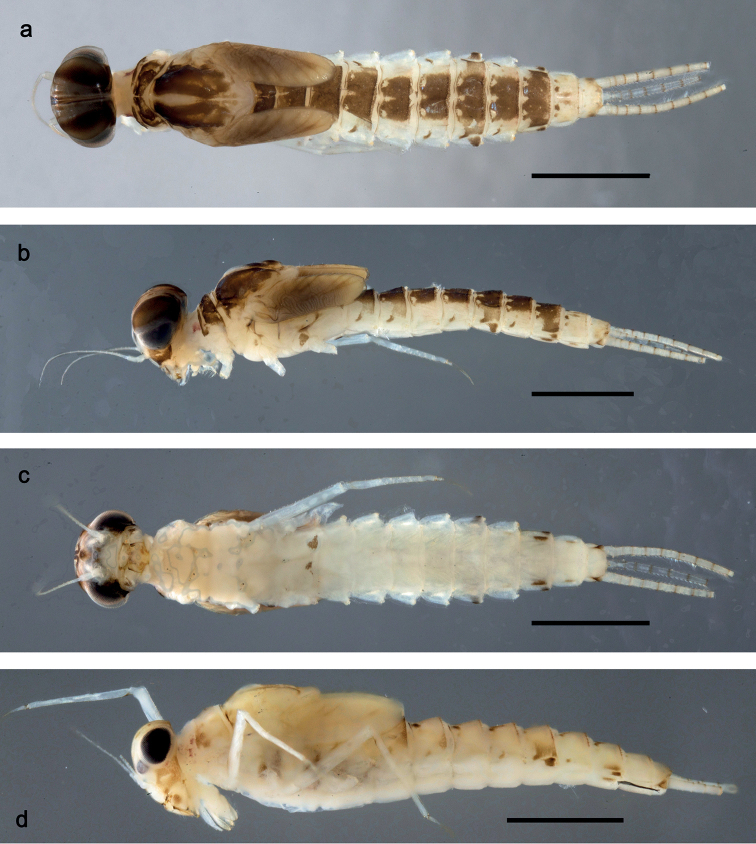
*Securiopsprimasia* sp. nov., larva habitus **a** male, dorsal view **b** male, lateral view **c** male, ventral view **d** female, lateral view (colour not yet fully developed) **a–c** mouthparts removed. Scale bars: 1 mm.

**Figure 2.**
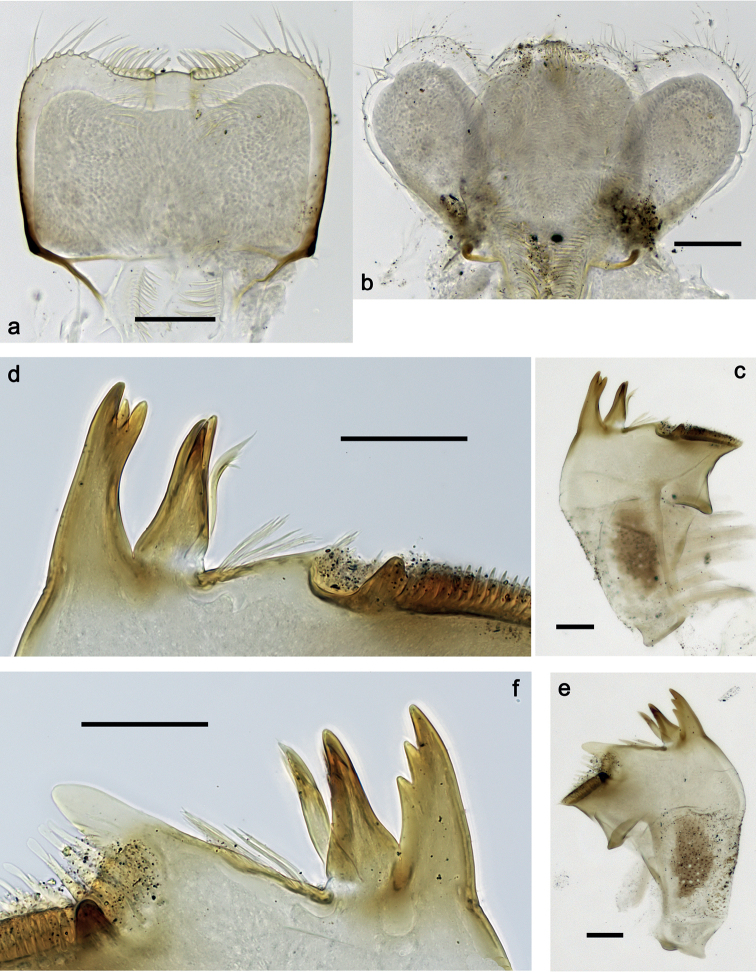
*Securiopsprimasia* sp. nov., larva morphology **a** labrum **b** hypopharynx and superlinguae **c, d** right mandible **e, f** left mandible. Scale bars: 50 µm.

**Figure 3.**
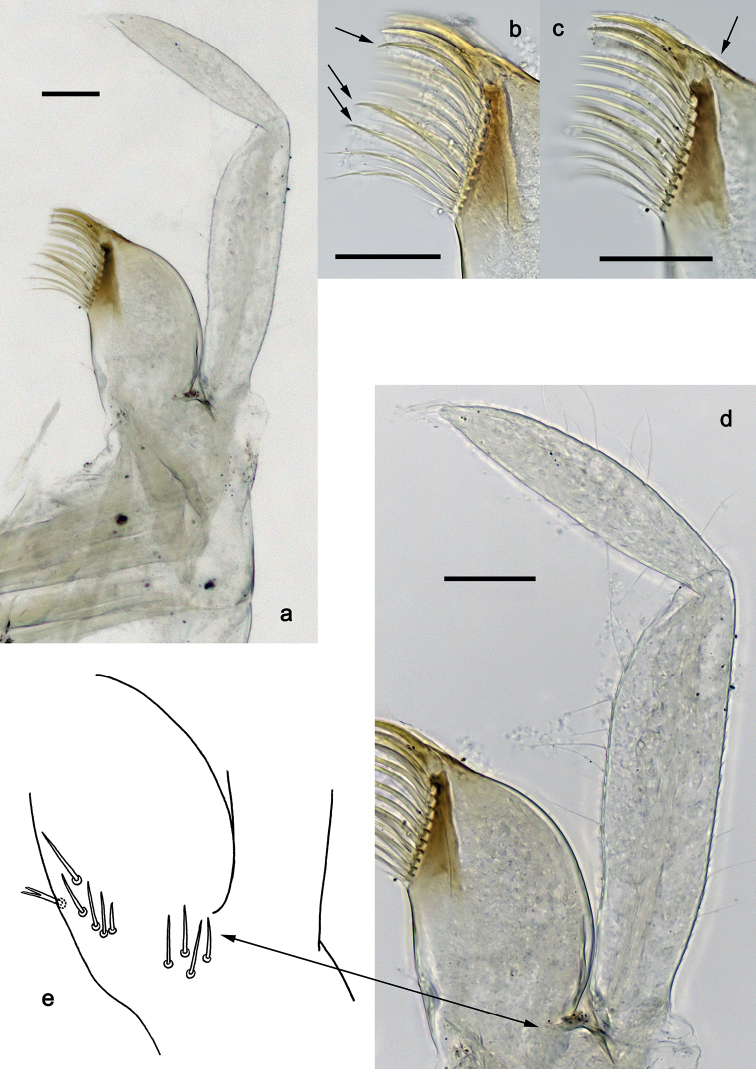
*Securiopsprimasia* sp. nov., larva morphology **a** maxilla **b** apex of maxilla, dorsal focus (arrows: denti-setae) **c** apex of maxilla, ventral focus (arrow: setae under canines) **d** maxillary palp **e** maxilla, middle part (ventrolateral view). Scale bars: 50 µm.

**Figure 4.**
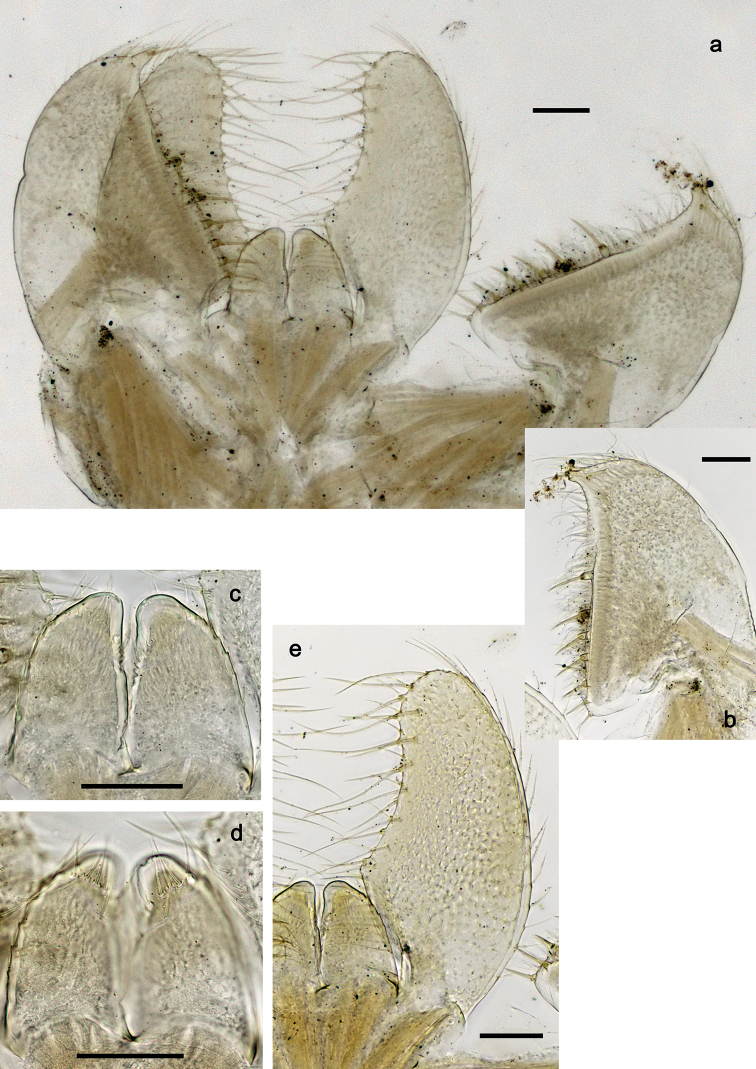
*Securiopsprimasia* sp. nov., larva morphology **a** labium **b** labial palp segment II **c** glossae (dorsal view) **d** glossae (ventral view) **e** paraglossa. Scale bars: 50 µm.

**Figure 5.**
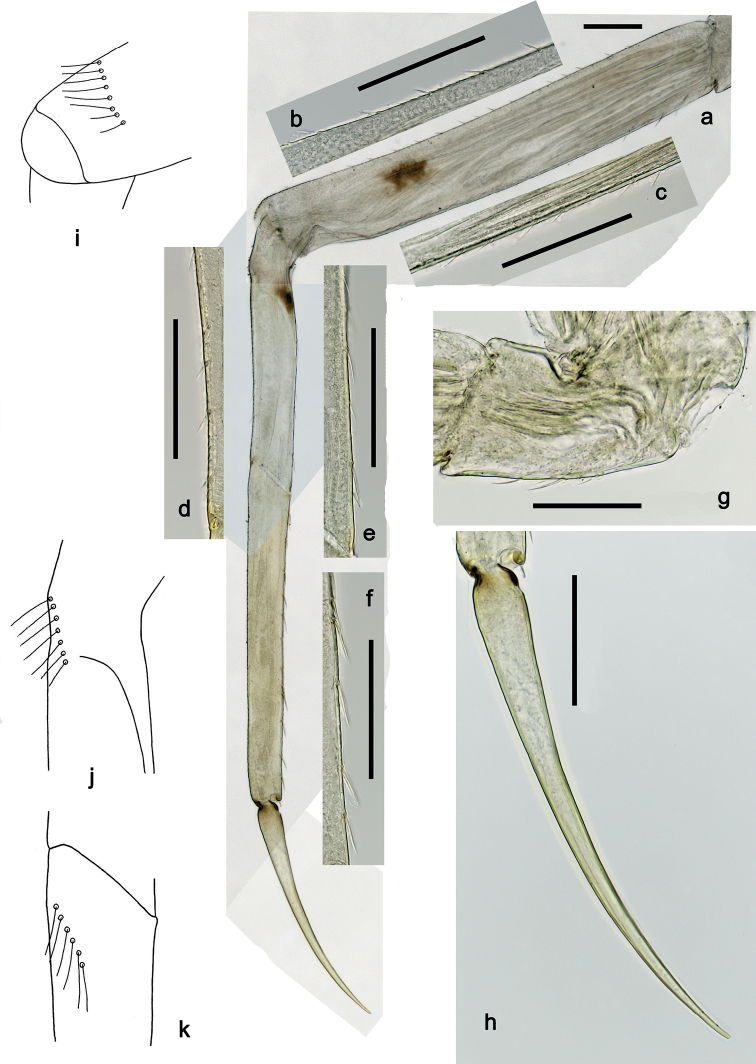
*Securiopsprimasia* sp. nov., larva morphology **a** foreleg **b** femur dorsal margin **c** femur ventral margin **d** tibia dorsal margin **e** tibia ventral margin **f** tarsus ventral margin **g** trochanter **h** claw **i** femur apex (posterior view) **j** tibia base **k** tarsus base. Scale bars: 100 µm.

**Figure 6.**
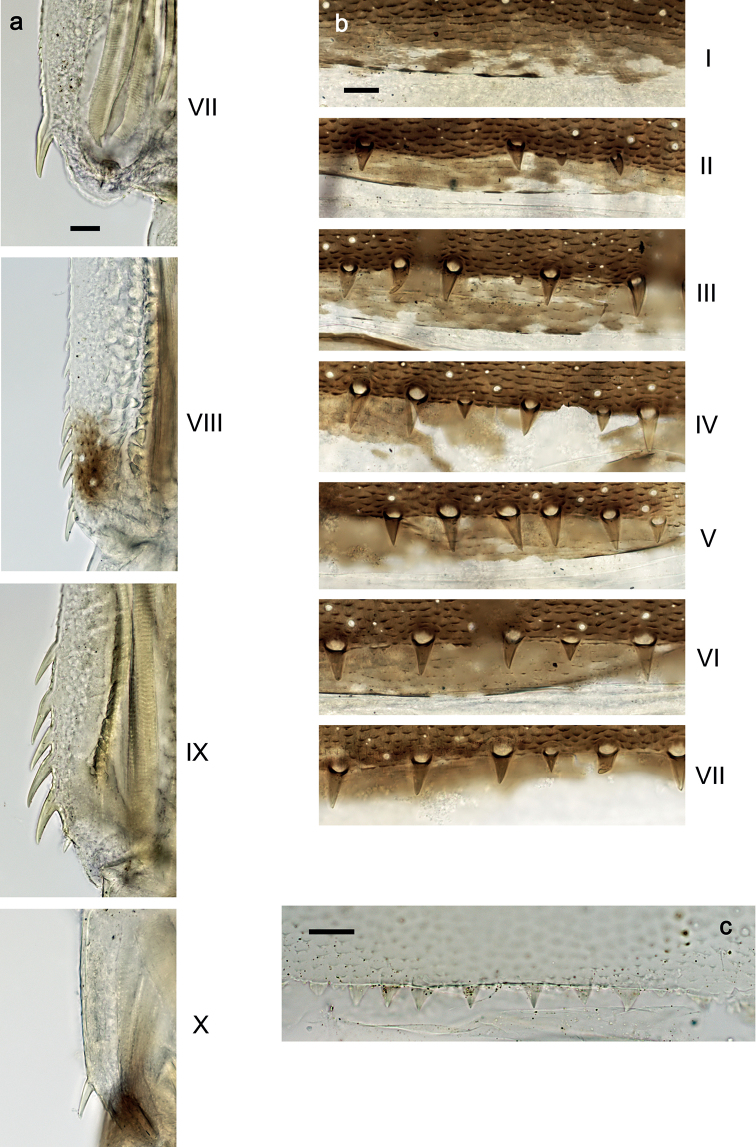
*Securiopsprimasia* sp. nov., larva morphology **a** abdominal terga VII–X, lateral margins **b** abdominal terga I–VII, posterior margins **c** abdominal sternum VI, posterior margin. Scale bars: 20 µm.

**Figure 7.**
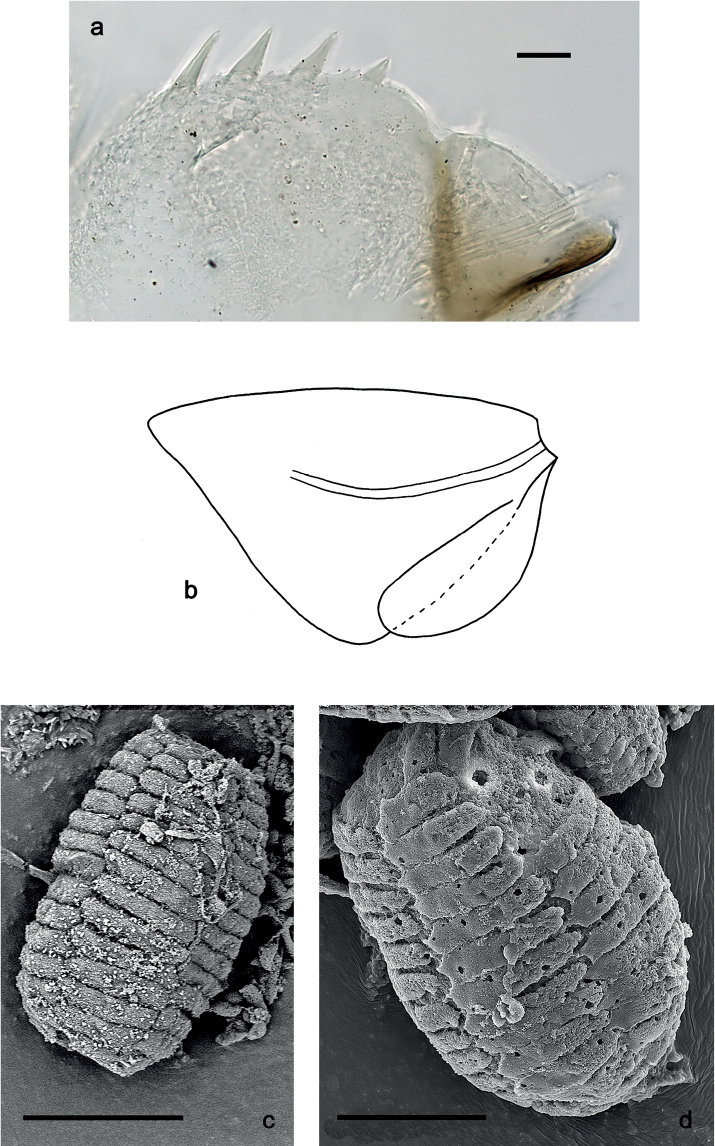
*Securiopsprimasia* sp. nov., morphology **a** paraproct **b** tergalius VII **c, d** eggs. Scale bars: 20 µm (**a**); 30 µm (**c, d**).

**Larva** (Figs 1–7). Body length 3.8–4.7 mm. Cerci approx. ½ body length, slightly longer than paracercus. Antennae somewhat longer than head length.

***Colouration*** (Figs 1a–d). Head, thorax and abdomen dorsally brown, with pattern as in Fig. 1a. Abdomen laterally light brown, with brown spots on most segments (absent or inconspicuous on segments I, IV and X), and larger additional brown spots on segments VI and VIII. Head, thorax and abdomen ventrally light brown, abdomen laterally with brown spots on segments VIII–X (Fig. 1c). Legs light brown, femur with distomedial brown spot, tibia with ventrobasal brown spot, and claw basally darker. Caudalii light brown, with brown annulation at distal margins of segments (Fig. 1a).

***Labrum*** (Fig. 2a). Rectangular, length ca. 0.7× maximum width. Distal margin with broad, shallow, medial emargination. Dorsal surface scattered with fine, simple setae; ventral surface with apicolateral patch of long, bifid setae near margin. Anterior margin apicolaterally with row of long, bifid setae, and medially with row of stout, medium, bifid setae.

***Right mandible*** (Fig. 2c, d). Incisor and kinetodontium cleft to base. Incisor with three denticles; kinetodontium with three denticles. Prostheca stick-like, apicolaterally denticulate. With restricted tuft of long setae between prostheca and mola. Tuft of setae at apex of mola present.

***Left mandible*** (Fig. 2e, f). Incisor and kinetodontium cleft to base. Incisor with three denticles; kinetodontium with four denticles. Prostheca stick-like, apicolaterally denticulate. With large tuft of long setae between prostheca and mola. Tuft of setae at apex of mola present.

***Hypopharynx and superlinguae*** (Fig. 2b). Lingua as long as superlinguae, broad; slightly longer than broad; distal margin almost straight, with fine, simple setae, not forming a medial tuft. Superlinguae distally broadly rounded; lateral margins rounded; fine, medium to long, simple setae along distal margin.

***Maxilla*** (Fig. 3a–e). Galea-lacinia ventrally with two simple, apical setae under canines (Fig. 3c). Canines long and very slender (Fig. 3a). Three long, slender, pectinate denti-setae (Fig. 3b). Medially with one bifid, spine-like seta (dorsolateral insertion) and two groups of simple, spine-like setae (ventrolateral insertions) (Fig. 3e). Maxillary palp 2-segmented, more than twice as long as length of galea-lacinia; palp segment II ca. 0.7× length of segment I; setae on maxillary palp long, fine, simple, scattered over surface of segments I and II; apex of last segment pointed (Fig. 3a, d).

***Labium*** (Fig. 4a–e). Glossa much shorter than paraglossa; outer margin with row of simple setae; inner margin distomedially serrate and with fine, simple setae; ventroapically with arc of long, simple setae. Paraglossa slightly curved inward; outer margin with row of long, simple setae; inner margin with medium row of simple setae, and submarginal row of long, simple setae, basally with dense row of long, simple setae. Labial palp 2-segmented. Segment II large, nearly trapezoidal with distal corner prolonged, pointed, curved inward; inner margin with many long, fine setae, in basal half with submarginal row of long, spine-like, setae-like processes; distal corner with dense, long, fine setae.

***Hind protoptera*** absent.

***Foreleg*** (Fig. 5a–k) very slender. Ratio of foreleg segments 2.0:1.0:1.4:0.7. ***Trochanter*.** Ventral margin with row of short, spine-like setae (Fig. 5g). ***Femur*.** Length ca. 6× maximum width. Dorsal margin with row of short, spine-like setae; distally with transverse arc of long, fine setae (difficult to see) (Fig. 5i). Apex rounded. Ventral margin with row of short, spine-like setae; femoral patch absent. ***Tibia*.** Dorsal margin with row of short, spine-like setae; proximally with arc of long, fine setae near margin (difficult to see) (Fig. 5j). Ventral margin with row of short to medium, curved, spine-like setae. Patellatibial suture present in basal 1/2 area. ***Tarsus*.** Dorsal margin bare; proximally with arc of long, fine setae near margin (difficult to see) (Fig. 5k). Ventral margin with row of medium, spine-like setae. ***Claw*** without denticles; subapical setae absent (Fig. 5h).

***Abdominal terga*** (Fig. 6a, b). Lateral margins of terga: VII with some minute spines; VIII with ca. seven small to large spines; IX with five large spines and one small spine (spine at posterolateral angle excluded from count); Posterior margins of terga: I smooth, without spines; II with strongly spaced or rudimentary, triangular spines; III–VII with spaced triangular spines, longer than wide.

***Abdominal sterna*** (Fig. 6c). Posterior margin of sterna: I–V smooth, without spines; VI–VIII with triangular spines.

***Tergalii*** (Fig. 7b). Present on segments I–VII; all tergalii with two lamellae, second lamella much smaller. Tracheae restricted to main trunk. Tergalius I as long as length of segment II; tergalius VII as long as length of segments VIII and half IX combined.

***Paraproct*** (Fig. 7a). With four larger, marginal spines, and some additional, minute spines in-between. Cercotractor with minute, marginal spines, hardly to see.

#### Imago.

Unknown.

#### Eggs

(Fig. 7c, d). Barrel-shaped, surface with four longitudinal rows of wide, sub-rectangular structural elements.

#### Genetics.

We obtained two sequences of 658 bp from specimens of two distinct populations. The K2P distance between them is 0.5%. The closest sequences available on GenBank and Bold system all belong to various species of *Cloeon* which is to be expected as no specimens of *Securiops* were previously sequenced.

#### Etymology.

Combination of the first part of the Latin word “prim-us” (meaning the first), and “asia” (for the continent), to highlight the first discovery of the Afrotropical genus *Securiops* in Asia.

#### Biological aspects.

The specimens were collected at altitudes between 100 m and 300 m.

#### Distribution

**Figure 8.**
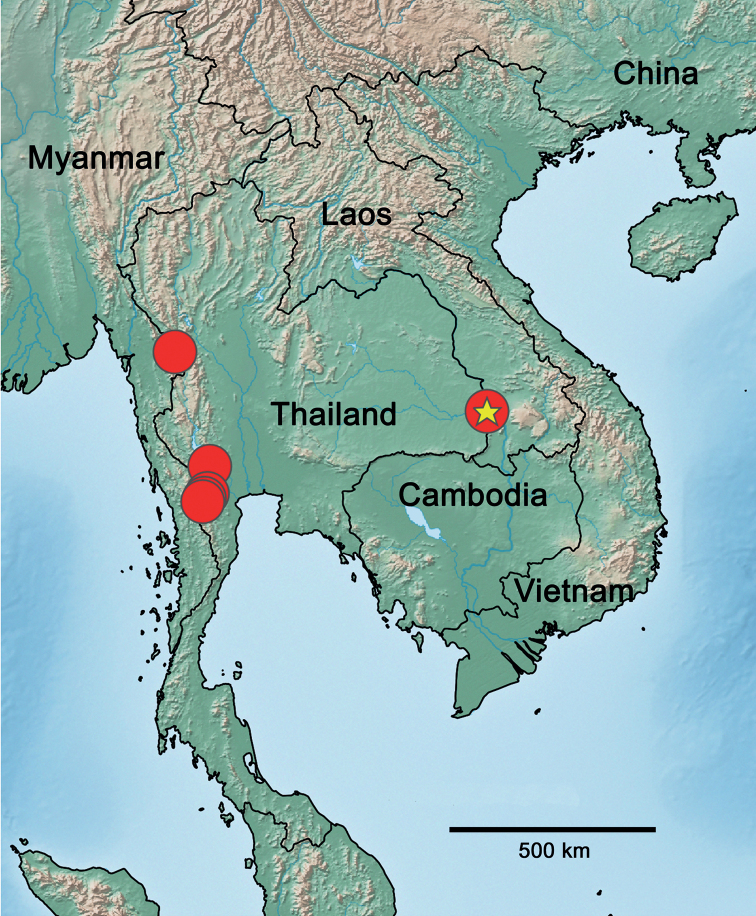
*Securiopsprimasia* sp. nov., distribution (yellow star: type locality).

**(Fig. 8).** Thailand.

#### Material examined.

**Type-material. *Holotype*.** Thailand • larva; Ubon Ratchathani Province, Khong Chiam District, Mekong River; 15°19'29"N, 105°30'07"E; 156 m; 21.v.2017; leg. S. Benjamas; on slide; GBIFCH00592671; KKU-AIC. ***Paratypes*.** Thailand • 3 larvae; Ubon Ratchathani Province, Khong Chiam District, Mekong River; 15°19'29"N, 105°30'07"E; 156 m; 21.v.2019; leg. S. Benjamas; 2 in alcohol; GBIFCH00975670; KKU-AIC; 1 on slide; GBIFCH00592672; MZL • 2 larvae; Ubon Ratchathani Province, Khong Chiam District, Mekong River; 15°19'29"N, 105°30'07"E; 156 m; 18.v.2017; leg. S. Benjamas; 2 on slides; GBIFCH00592670; MZL; GBIFCH00592669; KKU-AIC • 2 larvae; Ubon Ratchathani Province, Khong Chiam District, Mekong River; 15°19'29"N, 105°30'07"E; 156 m; 18.v.2017; leg. S. Benjamas; KKU-AIC.

#### Other material.

Thailand • larva; Kanchanaburi Province, Klong Ta Phoen; 14°06'54"N, 99°23'50"E; 31 m; 09.xi.2018; leg. C. Suttinun; in alcohol; GenBank OQ573687; GBIFCH00763772; KKU-AIC • larva; Tak Province, Huai Pu Ter; 16°37'51"N’, 98°37'44"E; 239 m; 27.xii.2017; leg. C. Suttinun; in alcohol; GenBank OQ573688; GBIFCH00763771; MZL • 3 larvae; Ratchaburi Province, Suan Phueng District, Pha Wo Thai; 13°30'56.1"N, 99°20'39.3"E; 118 m; 20.xi.2022; leg. C. Auychida; in alcohol; VMCMU • 2 larvae; Ratchaburi Province, Suan Phueng District, Kang Som Meow; 13°24'37.2"N, 99°16'37"E; 207 m; 20.xi.2022; leg. C. Auychida; in alcohol; VMCMU • 2 larvae; Ratchaburi Province, Suan Phueng District, Ton Nam Pha Chi; 13°20'11.2"N, 99°14'24.8"E; 265 m; 20.xi.2022; leg. C. Auychida; in alcohol; VMCMU.

## ﻿Discussion

### ﻿Assignment to *Securiops*

The new species clearly belongs to the genus *Securiops*, based on the following characters: (1) labrum rectangular, with a broad shallow, medial incision at the distal margin (Fig. 2a); (2) labium with strongly reduced glossae, enlarged paraglossae, and very broad, hatchet-like 2-segmented palps (Fig. 4a); (3) tergalii I–VII with two lamellae (Fig. 7b); (4) legs very elongate (Fig. 5a); (5) claws very elongate, without denticles (Fig. 5h); and (6) lateral margins of posterior abdominal segments with sharp spines (Fig. 6a) (Jacobus et al. 2006). Additionally, *S.primasia* sp. nov. shares with *S.megapalpus* the arcs of long, fine setae dorsodistally on the femur and dorsoproximally on the tibia and tarsus (Jacobus et al. 2006: figs 15, 16). This combination of characters and especially the shape of the labrum and labium clearly indicate the assignment to *Securiops* and not to other genera or subgenera closely related to *Procloeon* s.l.

### ﻿Differences to Afrotropical species of *Securiops*

Contrary to the Afrotropical species of *Securiops*, *S.primasia* sp. nov. has complete rows of short setae at the dorsal and ventral margins of the femur and tibia, and not just a few setae. Additional to the marginal setation of the legs, *S.primasia* sp. nov. can be differentiated from the Afrotropical species at least by the following main characters (Lugo-Ortiz and McCafferty 1996; Gattolliat 2003; Jacobus et al. 2006): (1) *S.megapalpus* with maxillary palp segment I much wider (1.9×) and much longer (2.6×) than segment II (*S.primasia* sp. nov. with segment I 1.6× width and 1.5× length of segment II); only tergalii I–IV with two lamellae (I–VII in *S.primasia* sp. nov.); tibia length 1.1× length of claw (1.4× in *S.primasia* sp. nov.); (2) *S.macafertiorum* with hind protoptera present (absent in *S.primasia* sp. nov); abdominal terga V–IX or VI–IX with spines on lateral margins (terga VIII and IX in *S.primasia* sp. nov.); (3) *S.mandrare* with spines on lateral margins of abdominal segments IV–IX (VIII and IX in *S.primasia* sp. nov.); tergalii on abdominal segments I-IV with two lamellae (I–VII in *S.primasia* sp. nov.); and (4) *S.mutadens* with spines on lateral margins of abdominal segments IV–IX (VIII and IX in *S.primasia* sp. nov.); tergalii on abdominal segments I-IV with two lamellae (I–VII in *S.primasia* sp. nov.).

### ﻿Eggs

The eggs of this genus are described for the first time. They present similarities with the eggs extracted from subimagos of *Baetisalpinus* Pictet, 1843, which should be a convergence (Fig. c, d; Kopelke and Müller-Liebenau 1982: fig. 6).

## ﻿Distribution

The occurrence of *Securiops* in Southeast Asia in addition to the Afrotropical region, where the four other known species live, is rare for Baetidae as well as for other families of Ephemeroptera. However, apart from the worldwide-distributed genus *Cloeon* Leach, 1815, there are other examples of Baetidae genera, for example, *Labiobaetis* Novikova & Kluge, 1987, *Nigrobaetis* Kazlauskas (in Novikova and Kluge), 1987, *Cheleocloeon* Wuillot & Gillies, 1993, and *Oculogaster* Kluge, 2016 (Barber-James et al. 2013; Kluge 2020a; Kaltenbach and Gattolliat 2021). The latter belongs to *Procloeon* s.l. as *Securiops* (Kluge 2020b). In other families, apart from the worldwide-distributed genus *Caenis* Stephens, 1835 (Caenidae), there are also genera with a distribution in the Afrotropical region as well as in the Oriental region (and mostly in the Palearctic as well), for example, *Ephemera* Linné, 1758 (Ephemeridae), *Afronurus* Lestage, 1924 (Heptagenidae), *Euthraulus* Barnard, 1932 (Leptophlebiidae), *Thraulus* Eaton, 1881 (Leptophlebiidae) and *Povilla* Navás, 1920 (Polymitarcyidae) (Barber-James et al. 2013). However, among the genera mentioned above, only *Oculogaster* and *Cheleocloeon* present a discontinuous distribution including exclusively Afrotropical and Oriental realms. Such a distribution pattern could be explained by a stepwise faunal exchange between Africa and Asia via corridors, which probably were more favourable for the dispersal of mayflies during some periods in the past, or by a fauna present on the Indian subcontinent before its drift to the north (Gattolliat and Nieto 2009).

*Securiops* in continental Africa, Madagascar and Thailand (present study) is rarely collected during standard protocols and freshwater surveys. It may be due to both its scarcity and its ecological requirements (probably partially psammophilous). We may expect that the occurrence of *Securiops* in Thailand is not an isolated distribution area. More collections in the yet poorly sampled Oriental region may lead to discoveries of a few more new species of *Securiops* in Southeast Asia, and maybe also on the Indian subcontinent.

## Supplementary Material

XML Treatment for Securiops primasia
